# Supplementary material for: Bionomics and population dynamics of anopheline larvae from an area dominated by fish farming tanks in northern Brazilian Amazon
Source: PLoS One. 2023 Aug 25;18(8):e0288983. doi: 10.1371/journal.pone.0288983 (PMC10456186; doi:10.1371/journal.pone.0288983)
Supplement: Attachment — Submitted filename: Specific response for each Reviewer.pdf [file pone.0288983.s001.pdf]

INPA
INSTITUTO NACIONAL DE PESQUISAS DA AMAZÔNIA

Ministério da Ciência, Tecnologia e Inovação

GOVERNO FEDERAL
BRASIL
PÁTRIA EDUCADORA

Manaus, April 5, 2023.

REBUTTAL LETTER

List of specific response of each Reviewer

Journal: **PLoS ONE**

Manuscript Number: **PONE-D-22-27653**

Title: Bionomics and population dynamics of anopheline larvae from an area dominated by fish farming tanks in northern Amazonian Brazil

Authors: Ledayane Mayana Costa Barbosa; Vera Margarete Scarpassa

Dr. Delfina Fernandes Hlashwayo

Academic Editor of PLOS ONE

Dear **Dr. Hlashwayo,**

We are very grateful to the you and two Reviewers for the comments and observations who significantly improved this manuscript. We hope that this revised version will be acceptable for publication in this important Journal.

We inform that the manuscript was extensively revised encompassing all sections, especially abstract. The title was also slightly modified as well as the English was improved throughout the manuscript, as are listed in “Revised manuscript with track copy”.

We also inform that Satellite image (**Figure 1**) was obtained from site https://eros.usgs.gov/# USGS EROS (Earth Resources Observatory and Science (EROS) Center) (public dominion) provided by you in the message “PLOS ONE Decision: Revision required [PONE-D-22-27653]”.

The **references** were revised in accordance the PLoS ONE’s Rules, as are listed in “Revised manuscript with track copy”.

We provided below a list of specific response for each Reviewer comment.

LIST OF SPECIFIC RESPONSES TO REVIEWER NUMBER 1:

Reviewer' comments #1:

Environmental parameters. The authors classified 3 breeding sites observed, namely: Fish farming tanks, natural ponds and stream (Figs 2A-C). Unfortunately, the vegetation that are feasible in the picture was not reported. Water vegetation is also important for some anopheles to avoid direct sun light in the tropical environment.

Author response: Sorry, we forget of include this information into first version of this manuscript. We provided this information "The aquatic vegetation found in breeding sites was identified as *Lemma* sp., *Pistia* sp., *Eichhornia* sp., *Nymphaea* sp. and *Juncus* sp. "This information is on page 12, lines 11 and 12.

Reviewer'comments #2:

Limnological parameter: In the Table 3, water salinity was not recorded. Any specific reason why salinity is not measured?

Author response:
We did not calculate the water salinity because the previous Limnological studies have reported that in the Amazon Delta and also for several tens of kilometers, along the coast, in north direction, salinity is close to zero. For this reason, we did not assess salinity in this study.

Reviewer'comments #2: Is the study site located far inland?

Author response: It is about 817 meters from Inland.

Reviewer'comments #3:
Entomological parameters: The "immature" stage is better replaced with larva(e).

Author response: We have attended this suggestion. However, there are few sentences into manuscript that we kept the word immature, because encompasses both larval and pupal stages. The pupae were also collected in this study and they were kept in plastic bottles with water from the breeding site until the emergence of adults and following were identified.

Reviewer'comments #4:

4. Results of this study underlines the importance of implementation of larval source management (LSM). As the majority of the breeding sites are fish farming tanks, the authors should discuss several options that might be suitable for the site and is acceptable by the owners of fish tank.

Author response: The ideal situation would be to use the fish farming tanks for the purpose for which they were built. However, the residents do not have the resources or funding from the government to carry out or to maintain this project. Then, the alternative situation and the most indicate and drastic, would be to neutralize or remove these tanks. This measure requires extensive discussion together with the Staffs of the Secretary of Health of the states and communities local. This is a very polemic situation. For this reason, we have limited this discussion into manuscript.

<u>LIST OF SPECIFIC RESPONSES TO REVIEWER NUMBER 2</u>:

<u>Reviewer'general comment</u>
This paper is well written. Though I have this few comments.

<u>Author response</u>:
Thank you

<u>Reviewer'comments #1:</u>

Rewrite abstracts should show problem and aim, methodology, result and conclusion. Write it clearly for a reader to understand the problem and the gap that you are trying to find the solution

<u>Author response</u>:
We have included these topics into abstract. Please, see page 2.

<u>Reviewer'comments #2:</u>

Introduction
When you say highest risk, you should show the number of incidences, and show how it lower the number of malaria cases, which interventions were used to lower interventions. Show the decrease in numbers.

<u>Author response</u>: We have attended this suggestion. These informations were included in first paragraph from Introduction section, page 3.

<u>Reviewer'comments #3:</u>

row number 1 material and methods You need to explain why you selected the study area. What were the reasons to select that area, was it because of higher malaria prevalence, was it because of agricultural activities that influence the presence of immature?

<u>Author response</u>: We have attended this suggestion. The sentence was included on page 5, lines 1, 2 and 3 from M&M section, as follow: "The selection of the study area took into account number of malaria cases, high incidence of *A. darlingi* and others vector [2, 7-9,18] and the high migratory flow of humans related to intense agricultural activities."

<u>Reviewer'comments #4</u>:

What were the limitations of this study? Need to explain the limitations so as to create the ground for those who will conduct this kind of study to take into consideration.

<u>Author response</u>: The difficulties found are common to other studies, such as limited or scarce funds (grants) and lack of field staff for help us. For this study, we had to pay a team to help us in field with our own salary. It was too hardy.

Vera Margarete Scarpassa
Corresponding Author
